# Exploring dual solutions and thermal conductivity in hybrid nanofluids: a comparative study of Xue and Hamilton–Crosser models

**DOI:** 10.1039/d3na00503h

**Published:** 2023-11-10

**Authors:** Mahnoor Sarfraz, Muhammad Yasir, Masood Khan

**Affiliations:** a Department of Mathematics, Quaid-i-Azam University Islamabad 44000 Pakistan myasir@math.qau.edu.pk

## Abstract

Hybrid nanofluids show great potential for heat transport applications such as solar thermal systems, car cooling systems, heat sinks, and thermal energy storage. They possess better thermal stability and properties compared to standard nanofluids. In this study, a base fluid, methanol, is injected into an electrically conducting heat-generating/absorbing disk of permeable boundary, and dual solutions are obtained. Two alternative models, Xue and Hamilton–Crosser are considered, and their thermal conductivities are contrasted. Furthermore, thermal radiation and ohmic heating are also considered, and convective boundary conditions are utilized to simulate overall heat gains or losses resulting from conduction, forced or natural convection between nearby objects of nearly constant temperature. Using a similarity transform, the governing equations are obtained and numerically solved *via* bvp4c, a finite difference method. It is observed that the presence of a magnetic field and the shrinking of the disk elevate the energy transport rate and wall stress. Additionally, the skin friction coefficient and thermal distribution rate increase with wall transmission constraint while fluid flow and energy transport diminish. Furthermore, particle clustering and nano-layer creation suggest that the Hamilton–Crosser model exhibits better thermal conductivity than the Xue model.

## Abstract 2

This study involves injecting a mixture of methanol and silica–alumina nanoparticles into an electrically conductive disk capable of generating or absorbing heat with a permeable boundary.

## Graphical abstract

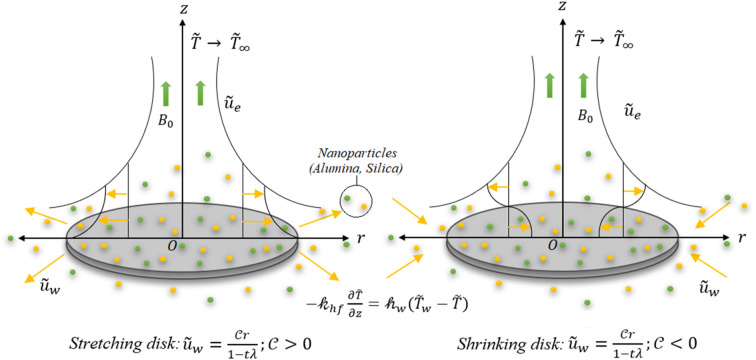

## 1. Introduction

Studies on nanofluid flow frequently prioritize the flow's thermal conductivity and heat transmission characteristics, while its thermophysical properties are often overlooked. However, including multiple nanoparticles in a base fluid can significantly increase the heat transfer phenomena. Hybrid nanofluids, composed of metal, polymeric, or non-metallic composite nanoparticles or a combination of different nanoparticles distributed in a base fluid, outperform traditional nanofluids with better pressure drop and heat transfer properties. The efficacy of ternary nanofluids is strongly influenced by the types, shapes, sizes, and percentages of the nanoparticles utilized. The revolutionary concept of carbon nanotubes was first introduced by Iijima^1^ through experimentation, resulting in the discovery of microscopic carbon layer straws known as carbon nanotubes. Although the essential components of the liquid were still complex hydrocarbon molecules, Choi and Eastman^2^ used the term “nanofluid” to describe fixed nanoscale particles floating in a fluid medium (nano-lubricants). Hybrid nanofluids possess exceptional mechanical, electrical, and thermal properties, as well as high electrical and thermal conductivities, making them strong and lightweight. Several studies on the applications of hybrid nanofluids are addressed in ref. 3–11. Moldoveanu *et al.*^12^ scrutinized the thermal conductivity of two nanofluids and their hybrid, and the experimental outcomes were presented at various temperatures and volume fractions, including room temperature.

Convective heat transfer models can present non-linearity issues and dual solutions, which can result in complex behaviors. Therefore, it is essential to understand both stable and unstable states. To address this, several studies have been conducted on different scenarios. For instance, Zheng *et al.*^13^ explored the radiation effect on velocity and temperature fields in a quiescent micropolar fluid with nonlinear power-law surface velocity and temperature distributions. In a similar study, Mahapatra *et al.*^14^ investigated dual solutions in magnetohydrodynamic stagnation-point flow and heat transfer over a shrinking surface with partial slip, while Rostami *et al.*^15^ developed an analytical solution for the steady laminar MHD mixed convection boundary layer flow of a SiO_2_–Al_2_O_3_/water hybrid nanofluid near the stagnation point on a vertical flat plate. Moreover, Mousavi *et al.*^16^ studied the dual solutions for Casson hybrid nanofluid flow due to a stretching/shrinking sheet with suction, radiation, and convective boundary condition effects. Asjad *et al.*^17^ introduced a new fractional operator to model memory effects and solved analytically for temperature and velocity fields using the Laplace transform approach. Studies of heat transport are discussed in further studies, as reported in ref. 18–28.

Drawing inspiration from the aforementioned studies, it is formulated that the premise that the base fluid, methanol (CH_3_OH), is comprised of a blend of silica (SiO_2_) and alumina (Al_2_O_3_), forming a disk with a porous boundary capable of generating and absorbing heat. The problem's coordinates take the form of a cylinder (*r*,*θ*,*z*), with the thermal conductivities of the two alternative models: Xue and Hamilton–Crosser, compared. The surface is seen to radially expand and contract with time, with convective boundary conditions provided. A uniform magnetic field is applied perpendicularly to the *z*-axis, with minimal interference from the electric field. Furthermore, both thermal radiation and ohmic heating effects are taken into consideration. The dimensionless equations are obtained through similarity profiles and numerically solved using bvp4c. The novelty of this study is in using these nanomaterials and the effects taken. Methanol-based hybrid nanofluids incorporating silica and alumina nanoparticles have demonstrated a significant increase in heat transport efficiency, attributed to their high thermal conductivities and extensive surface area. These hybrid nanomaterials have garnered attention for superior rheological, thermal, and economic performance compared to monotype nanofluids. They enhance convective heat transfer, reduce boundary layer thickness, and provide thermal stability, making them indispensable for diverse applications such as solar thermal systems, car cooling, and heat sinks.

The article is structured into four distinct sections. In Section 2, the mathematical problem at hand is comprehensively addressed, providing a detailed insight into the complex computations and analytical techniques employed to obtain the desired results. Section 3 is devoted to graphical illustrations, accompanied by their corresponding physical significance, which helps readers visualize the intricate details of the problem and facilitates better understanding. Finally, Section 4 presents a concise summary of the findings, highlighting the key takeaways from the study and providing insights for future research in this area.

## 2. Problem formulation

In this study, hybrid nanofluids consisting of silica and alumina nanoparticles in methanol are considered, which have been shown to exhibit excellent cooling properties. The disk's boundary is assumed to be porous, allowing heat generation and absorption. The problem is analyzed using cylindrical coordinates (*r*,*θ*,*z*), and the velocity field **V** is characterized by the components [*ũ*(*r*,*t*), *w̃*(*r*,*t*)]. The thermal conductivity of two models is considered, namely Xue and Hamilton–Crosser. The surface of the disk is assumed to be radially expanding or contracting, which leads to a velocity component along the boundary of 
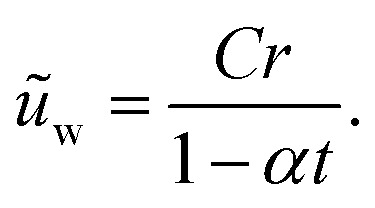
 In the far-field region near the stagnation point, the velocity is given by 
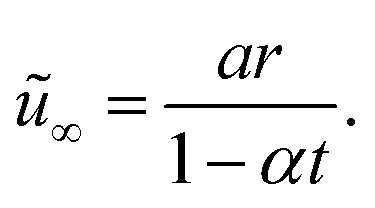
 A uniform magnetic field **B** = [0, 0, *B*_0_] is applied perpendicular to the *z*-axis, and the effects of the electric field are assumed to be negligible. The study also considers the effects of ohmic heating and thermal radiation. The surface of the disk is heated by convection from a hot fluid with temperature *T̃*_w_ and heat transfer coefficient *h*_w_, while the ambient temperature distribution is represented by *T̃*_∞_.

**Fig. 1:**
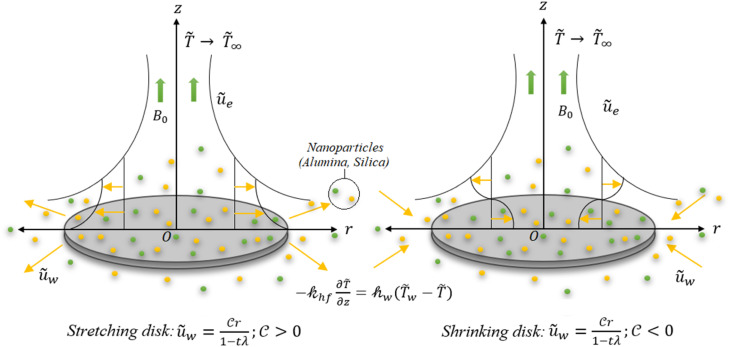
Flow mechanism.

To assist in understanding, Fig. 1 displays the geometry of the problem, while Table 1 presents the thermophysical characteristics of both the methanol and alumina–silica nanoparticles.

**Table 1:** Numerical values for the thermophysical features

Physical properties	Base fluid	Nanoparticles	
	CH_3_OH	SiO_2_	Al_2_O_3_
*ϱ*_f_	792	2650	3970
*c*_p_	2545	730	765
*k*	0.2035	1.5	40
Pr at 25 °C	6.83	—	—
*s*	3.7 (spherical)		

The governing equations are (see ref. 16)1
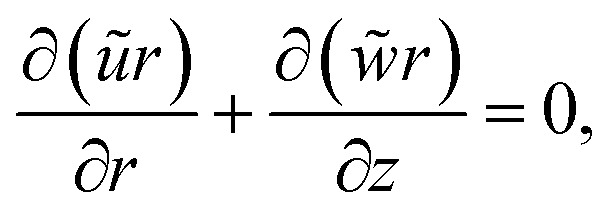
2
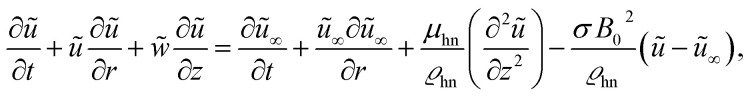
3
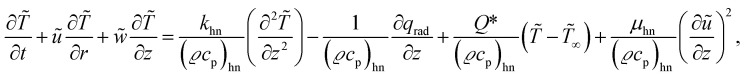
where 
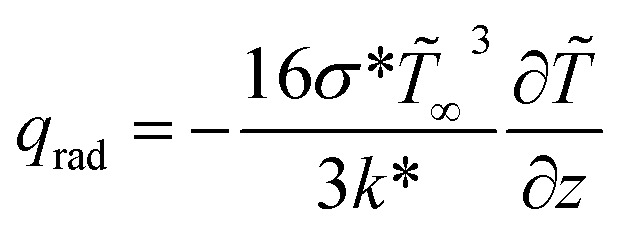
 is radiative heat flux.

The thermophysical properties are given as (see ref. 7)4
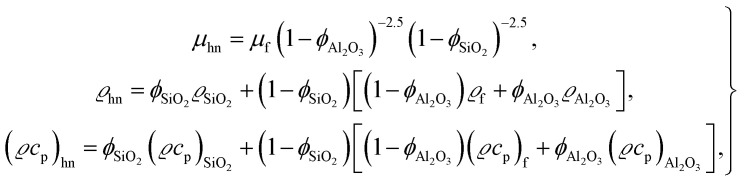


For Hamilton–Crosser model, the thermal conductivity is given as5
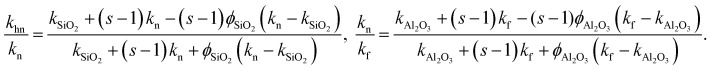


The value of *s* vary for different shapes, such as for spherical *s* = 3.0. For Xue model, the thermal conductivity is given as6
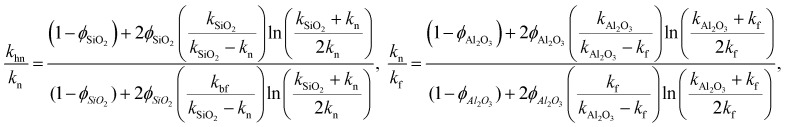


The boundary conditions (BCs) are (see ref. 16)7
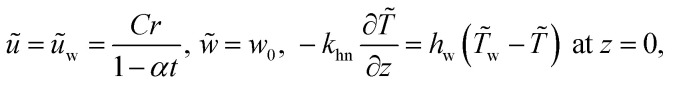
8
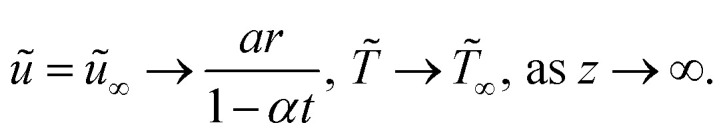


Introducing stream function 
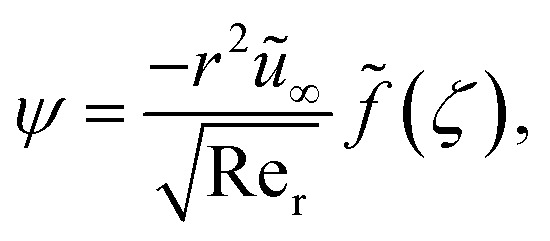
 where 
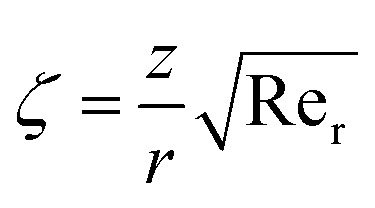
 and local Reynolds number 
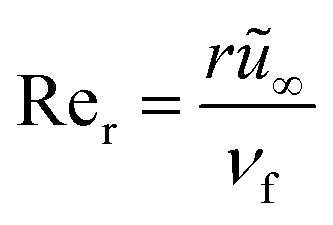
 with the following similarity ansatz9
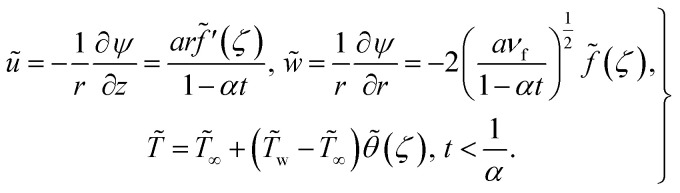


The negative sign in eqn (9) indicates that the fluid at the far-field is being pulled towards the surface, rather than being pushed away from it. Using eqn (11)10
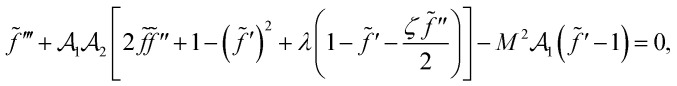
11
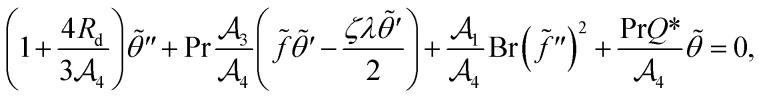
12
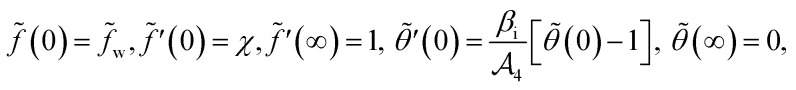
where for ternary hybrid nanofluids13
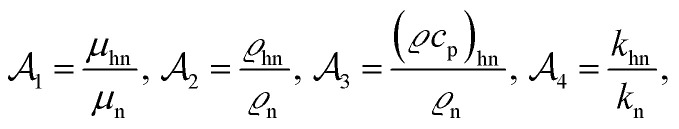


The dimensionless parameters are14
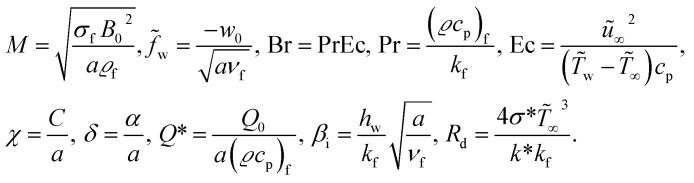


The quantities of engineering interest, *C̃*_f_ and Nu_r_ are given as15
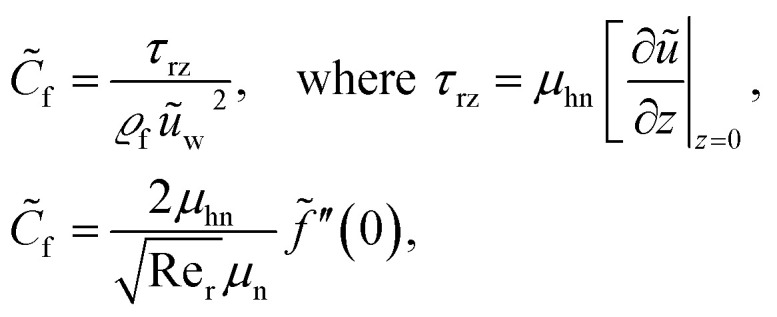
16
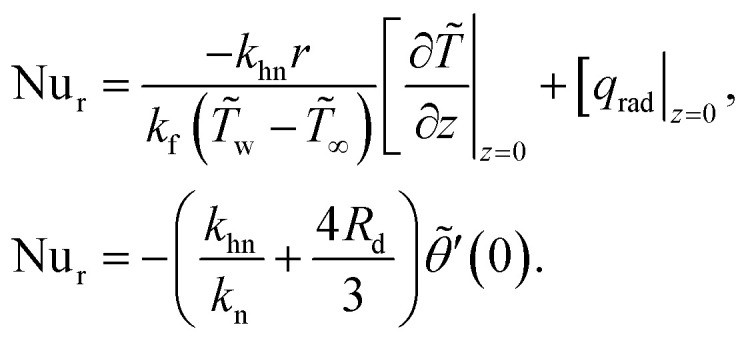


## 3. Interpretation and analysis of results

This section presents an analysis of the numerical results obtained for various relevant parameters, such as velocity and temperature distributions, and the effects of control parameters on Nu_r_ and *C̃*_f_. This study presents the solution of boundary value problems (BVPs) for ordinary differential equations using MATLAB's bvp4c function. Our approach is based on using a finite difference method to discretize the BVP, which allows us to solve the resulting system of equations using a collocation method. To achieve the desired level of accuracy, the number of mesh points is adjusted, and the initial guess is provided to the solver. The estimated error count is constantly monitored during the computation, ensuring the quality of the solution. During the calculations, some parameters are held constant, and the behavior of the first (stable) and second (unstable) solutions is examined. The considered values and ranges of the parameters are Re = 0.6, 1 ≤ Pr ≤ 10, 1 ≤ *β*_i_ ≤ 3, 0.01 ≤ δ* ≤ 0.05, 0.5 ≤ *f̃*_w_ ≤ 4, 0.01 ≤ Ec ≤ 0.08, 0.01 ≤ *M* ≤ 2, and 0.1 ≤ *λ* ≤ 2, and spherical shaped nanoparticles are used. The behavior of a mixture of nanoparticles, namely silica and alumina, submerged into a solution of methanol, *i.e.*, (SiO_2_–Al_2_O_3_/CH_3_OH), is examined for two different models, Xue and Hamilton–Crosser models. Solid lines represent the behavior of the first solution, while dot-dashed lines depict the second solution.

**Fig. 2:**
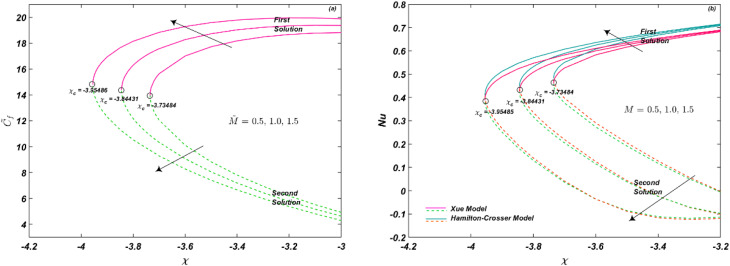
(a and b) Influence of *M* on *C̃*_f_ and Nu_r_.

Fig. 2(a) and (b) elucidate the impact of the magnetic field parameter *M* on the key parameters, namely *C̃*_f_ and Nu_r_. Both figures represent the first and second solutions for control parameters *χ* and *M* = 0.5, 1.0, 1.5. Fig. 2(a) demonstrates that both solutions' values of *C̃*_f_ escalate with an increase in *M*. The critical values, *χ*_c_ = −3.73484, −3.84431, −3.95486, are observed at *M* = 0.5, 1.0, 1.5, respectively. This enhancement in the behavior of *C̃*_f_ can be attributed to the linear increment in *C̃*_f_ with the magnetic field's increase. The magnetic field induces Lorentz forces that alter the velocity profile of the fluid, resulting in changes to the skin friction coefficient. Specifically, the skin friction coefficient decreases with an increasing magnetic field due to the development of a boundary layer with lower momentum. The Lorentz force causes the flow field to decline, magnifying the frictional forces, thus augmenting *C̃*_f_. On the other hand, Fig. 2(b) demonstrates the impact of *M* on Nu_r_, which indicates that heat energy gains result from an increase in magnetic force, leading to chaotic particle motion. The system temperature increases due to the fluid molecules' physical energy and their greater intermolecular vibrations caused by increased kinetic energy. The magnetic field enhances fluid mixing and promote heat transfer, leading to an increase in the Nusselt number. It is noteworthy that the behavior of Xue and Hamilton–Crosser models is depicted here. The results indicate that the magnetic parameter has improved the outcomes of Nu_r_. Moreover, the values for the Hamilton–Crosser models are more pronounced than the Xue model, indicating that they have better thermal conductivity than the Xue model. The critical values, *χ*_c_ = −3.73484, −3.84431, −3.95486, are observed at *M* = 0.5, 1.0, 1.5, respectively.

**Fig. 3:**
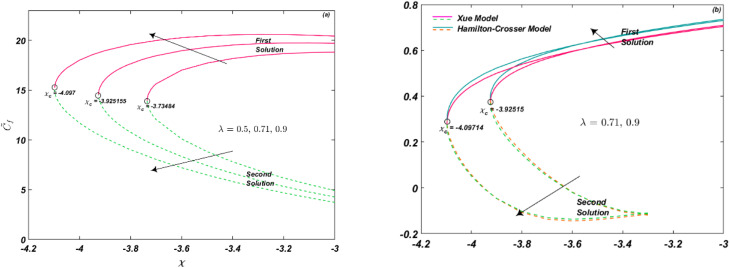
(a and b) Influence of *λ* on *C̃*_f_ and Nu_r_.

Fig. 3(a) and (b) demonstrate the influence of unsteadiness parameter *λ* on *C̃*_f_ and Nu_r_, respectively. Critical values *χ*_c_ = −4.097, −3.925155, −3.73484 at *λ* = 0.5, 0.71, 0.9, respectively, are observed. As depicted in Fig. 3(a), an increase in *λ* leads to an enhancement in *C̃*_f_ (for both solutions), resulting in a linear increment over time. Physically, unsteadiness can cause a delay in fluid flow, leading to an increase in frictional forces. Conversely, Fig. 3(b) illustrates the impact *λ* on Nu_r_, with critical values of *χ*_c_ = −4.097, −3.925155 at *λ* = 0.5, 0.71, respectively. Augmentation in thermal transport rate occurs as a result of an increase in unsteadiness, which leads to chaotic particle motion, greater intermolecular vibrations, and increased kinetic energy, resulting in elevated system temperatures. The behavior of Xue and Hamilton–Crosser models is depicted for first and second solutions, with Hamilton–Crosser models exhibiting better thermal conductivity than the Xue model.

**Fig. 4:**
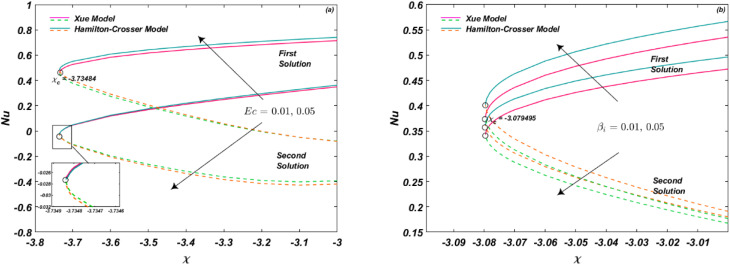
(a and b) Influence of Ec and *β*_i_ on Nu_r_.

Fig. 4(a) and (b) depict the influence of Eckert number and Biot number on Nu_r_. It can be observed that Nu_r_ increases with an increase in Ec, which represents the self-heating of the fluid due to dissipation effects and temperature gradients. Notably, the critical value remains the same for different values of Ec, *i.e.*, *χ*_c_ = −3.73484 for Ec = 0.01, 0.05. On the other hand, the Biot number *β*_i_ relates the rate of heat transfer inside a solid to the rate of heat transfer at the solid's surface. It is defined as the ratio of the internal thermal resistance to the external thermal resistance. Fig. 4(b) demonstrates that an increase in *β*_i_ leads to an increase in thermal transport rate. The critical value is *χ*_c_ = −3.079495 for *β*_i_ = 0.01, 0.05. In general, the Nu_r_ increases with increasing Biot number. This is because as the *β*_i_ increases, the thermal resistance of the solid becomes smaller compared to the thermal resistance of the fluid, and the temperature gradient in the fluid becomes more uniform. This leads to increased heat transfer from the solid to the fluid, and therefore a higher convective heat transfer coefficient, resulting in a higher Nu_r_.

**Fig. 5:**
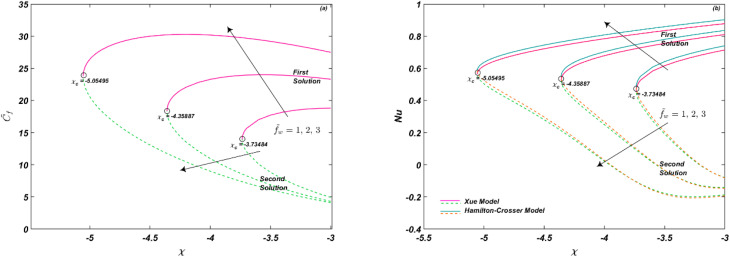
(a and b) Influence of *f̃*_w_ on *C̃*_f_ and Nu_r_.

**Fig. 6:**
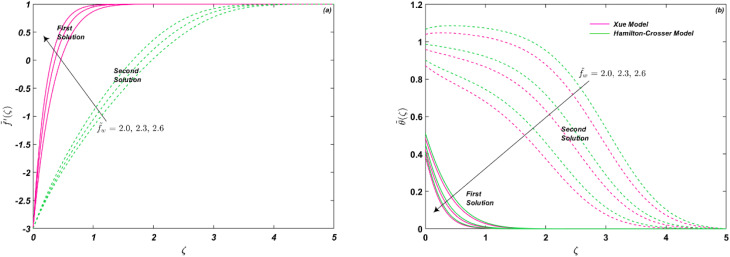
(a and b) Influence of *f̃*_w_ on *f̃*′(*ζ*) and *θ̃*(*ζ*).

The impression of *f̃*_w_ is noted in Fig. 5(a) and (b) on *C̃*_f_ and Nu_r_, respectively. The mass transmission constraint shows the effect of suction (*f̃*_w_ > 0) and injection (*f̃*_w_ < 0). When *f̃*_w_ > 0, fluid suction at the surface lowers the viscous effects closer to the wall. Suction has the potential to lower fluid velocity *f̃*′(*ζ*), whereas blowing or injection has the opposite effects. As a result, greater suction velocities yield higher entertainment velocities. Thus, suction causes the fluid's velocity to decrease, while blowing causes it to increase, as shown in Fig. 6(a). Therefore, *C̃*_f_ improves for *f̃*_w_ > 0, as shown in Fig. 5(a). On the other hand, energy of the system is declined as given in Fig. 6(b). However, Fig. 5(b) depicts that Nu_r_ is an increasing function of *f̃*_w_ as well. It is seen that the thermal conductivity of Hamilton–Crosser's model is more than as compared to Xue's model. The critical values for Fig. 5(a) and (b) are *χ*_c_ = −3.73484, −4.35887, −5.05495 at *f̃*_w_ = 1, 2, 3, respectively.

**Fig. 7:**
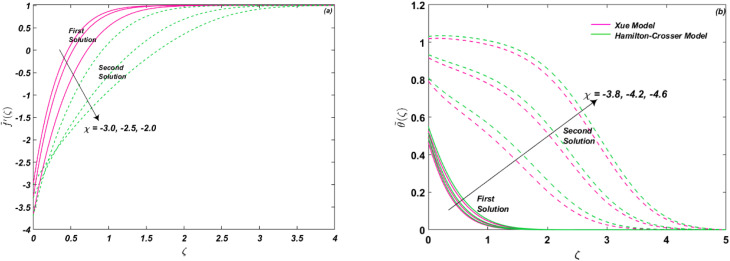
(a and b) Influence of *χ* on *f̃*′(*ζ*) and *θ̃*(*ζ*).

The behavior of shrinking/stretching parameter *χ* is noted in Fig. 7(a) and (b) on flow and energy distribution. The parameter shows the effect of stretching (*χ* > 0) and shrinking (*χ* < 0). It is illustrated that shrinking causes the flow field to decline for both solutions, as shown in Fig. 7(a). However, the temperature profile is boosted significantly for both models, as given in Fig. 7(b). Whereas it is seen that due to particle clustering and nano-layer creation, the thermal conductivity of Hamilton–Crosser is better than Xue model.

**Fig. 8:**
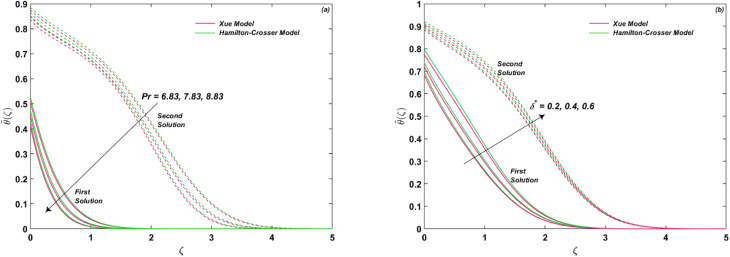
(a and b) Influence of Pr and δ* on *θ̃*(*ζ*).

Fig. 8(a) and (b) provide an insight into the impact of Prandtl number Pr and heat generation/absorption parameter δ* on the system's behavior. The Prandtl number is a ratio of momentum to thermal diffusivity, indicating the dominance of thermal diffusion mechanism for a given fluid. The value of Pr for methanol at 25 °C is 6.83, much higher than that of air (Pr = 0.71). A lower value of Pr signifies the prevalence of heat conduction over convection, where heat diffuses faster than fluid velocity. In general, Pr for gases ranges around 0.7, while it varies between 1 and 10 for fluids. Fig. 8(a) indicates that the thermal transport decreases with increasing Pr, implying that a higher Pr value leads to a significant reduction in temperature/energy transport. The presence of a heat source or sink has a significant effect on the temperature distribution in a system. A heat source increases the temperature in its vicinity, while a heat sink reduces it. This effect propagates through the system *via* heat transfer mechanisms such as conduction, convection, and radiation. The magnitude of the effect depends on the strength and location of the heat source or sink, as well as the thermal properties and boundary conditions of the system. The effect of a heat source or sink can be used to control or optimize temperature distribution in a system *θ̃*(*ζ*), as shown in Fig. 8(b).

By contrasting the existing numerical values *C̃*_f_ with the prior results, the current model in Table 2 is validated. The variation of *χ* < 0 is presented in the absence of nanoparticles and magnetic field. It is seen that the coefficient of skin friction enhanced significantly due from *χ* = −0.25 to −0.95.

**Table 2:** Comparison of *C̃*_f_ for various values of *χ* in the absence of nanoparticles and magnetic field

*χ*	Khan *et al.*^30^	Wang^29^	Present study
			First solution
−0.25	1.4566365	1.456640	1.456640
−0.5	1.4900104	1.490010	1.490041
−0.75	1.3528399	1.352840	1.352838
−0.95	0.9469034	0.946900	0.946904

### 3.1 Stability analysis

**Fig. 9:**
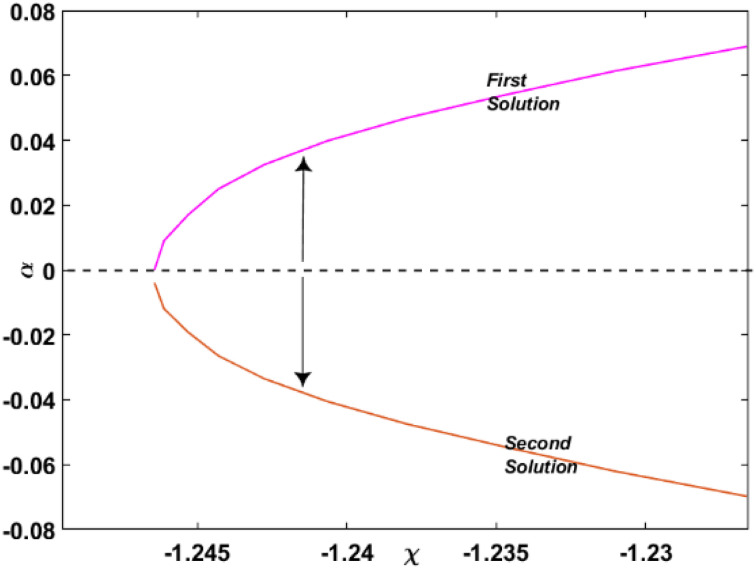
Smallest eigenvalues *α* for *χ*.

The current research used a stability analysis to validate the obtained solution. This is of utmost significance, mainly when the governing system permits multiple-branch solutions. Identifying all probable solutions emerging from the governing boundary layer problems is essential for determining the solution. Based on previous literature studies,^31–35^ the smallest eigenvalues *α* were plotted against *χ*, and the resulting Fig. 9 was obtained. The physical interpretation of this figure is that a positive value of *α* indicates an initial deterioration of disturbance, implying that the flow is in a stable mode. Conversely, a negative value of *α*, as *τ* → ∞, implies that the flow is in an unstable state due to the early increase of disturbance. It should be noted that as *χ* approaches the critical value, *χ*_c_ = −1.2431, both the stable and unstable branches converge to *α* = 0. This behavior indicates that the solutions bifurcate at the critical values, and it has critical implications for the study of boundary layer problems.

## 4. Conclusion

An electrically conducting heat-generating/absorbing disk was assumed with a porous boundary immersed in a mixture of silica (SiO_2_) and alumina (Al_2_O_3_) into methanol (CH_3_OH) which acts as a base fluid. The thermal conductivity of two models, Xue and Hamilton–Crosser were considered and compared. The effects of MHD, heat source/sink, suction/injection, thermal radiation, and convective boundary conditions were scrutinized over a radially shrinking/stretching surface. The primary outcomes are given below.

❖ Due to particle clustering and nano-layer creation, the thermal conductivity of the Hamilton–Crosser was better than the Xue model.

❖ Energy transport rate and wall stress were elevated due to shrinking and the presence of the magnetic field.

❖ Unsteadiness declined the fluid motion, which caused the frictional forces to incline.

❖ Due to the increment in Biot number, the surface heat resistance declined, which dominated the convection mechanism resulting in a higher thermal transport rate.

❖ The critical value was the same for variation of Eckert and Biot numbers, *i.e.*, *χ*_c_ = −3.73484 and −3.079495, respectively.

❖ Coefficient of skin friction and thermal distribution rate was an increasing function of wall transmission constraint, whereas the fluid flow and energy transport diminished.

❖ Incrementing Prandtl number reduced the temperature and energy transport significantly.

❖ The thermal transport was dominant due to the heat generation/absorption parameter.

## Nomenclature

(*ũ*,*ṽ*,*w̃*): Velocity components (m s^−1^)
*k*
: Thermal conductivity (W m^−1^ K^−1^)
*ν*
: Kinematic viscosity (m^2^ s^−1^)
*ϱ*
: Density (kg m^−3^)
*p̃*
: Pressure (N m^−2^)
α: Unsteadiness rate (s^−1^)
μ: Absolute viscosity (kg m^−1^ s^−1^)
σ: Electric conductivity (Ω m)
*B*_0_
: Magnetic field strength (N s C^−1^ m^−1^)
Re_r_: Reynolds number
σ*: Stefan–Boltzmann coefficient
*h*_w_
: Heat transfer coefficient
*τ*_w_
: Wall-shear stress
*T̃*
: Temperature (K)
*f̃*′,** Dimensionless velocity and temperature
*f̃*_w_
: Wall mass transfer coefficient
*λ*
: Unsteadiness parameter
*p̃*
: Dimensionless pressure
*M*
: Magnetic field parameter
*γ*_1_
: Biot number
*R*_d_
: Radiation parameter
*ϕ*
: Nanoparticle volume fraction
*ζ*
: Dimensionless variable
k*: Mean absorption coefficient
Pr: Prandtl number
Nu_r_: Nusselt number

### Subscripts

r,z: Derivative w.r.t r and z
∞: Far-field condition
w: Wall boundary condition
f: Base fluid
n: Nanofluid
hn: Hybrid nanofluid

### Superscripts

′: Derivative w.r.t *ζ*

## Conflicts of interest

There are no conflicts to declare.

## Supplementary Material

## References

[cit1] Iijima S. (1991). Helical microtubules of graphitic carbon. Nature.

[cit2] Choi S. U. and Eastman J. A., Enhancing thermal conductivity of fluids with nanoparticles, Developments and Applications of Non-Newtonian Flows, FED-vol. 231/MD-vol. 66, 1995, pp. 99–105

[cit3] Alkasasbeh H., Swalmeh M., Bani Saeed H., Al Faqih F., Talafha A. (2020). Investigation on CNTs-water and human blood based Casson nanofluid flow over a stretching sheet under impact of magnetic field. Front. Heat Mass Transfer.

[cit4] Sheikholeslami M. , Abohamzeh E., Ebrahimpour Z. and Said Z., Brief overview of the applications of hybrid nanofluids, Hybrid Nanofluids, 2022, pp. 171–202

[cit5] Kazemian A., Salari A., Ma T., Lu H. (2022). Application of hybrid nanofluids in a novel combined photovoltaic/thermal and solar collector system. Sol. Energy.

[cit6] Smaisim G. F., Abdulhadi A. M., Uktamov K. F., Alsultany F. H., Izzat S. E., Ansari M. J., Kzar H. H., Al-Gazally M. E., Kianfar E. (2022). Nanofluids: properties and applications. J. Sol-Gel Sci. Technol..

[cit7] Yasir M., Khan M., Sarfraz M., Abuzaid D., Ullah M. Z. (2022). Exploration of the dynamics of ethylene glycol conveying copper and titania nanoparticles on a stretchable/shrinkable curved object: Stability analysis. Int. Commun. Heat Mass Transfer.

[cit8] Sarfraz M., Khan M. (2022). Significance of ethylene glycol-based CNT Homann nanofluid flow over a biaxially stretching surface. Waves Random Complex Media.

[cit9] Sarfraz M., Khan M. (2023). Thermodynamic irreversibility analysis of water conveying argentum and titania nanoparticles subject to inclined stretching surface. Phys. Scr..

[cit10] Yang Y., Zheng S., Lu Q. (2021). Numerical solutions of non-gray gases and particles radiative transfer in three-dimensional combustion system using DRESOR and SNBCK. Int. J. Therm. Sci..

[cit11] Yasir M., Khan M., Alqahtani A. S., Malik M. Y. (2023). Heat generation/absorption effects in thermally radiative mixed convective flow of Zn–TiO2/H2O hybrid nanofluid. Case Stud. Therm. Eng..

[cit12] Moldoveanu G. M., Huminic G., Minea A. A., Huminic A. (2018). Experimental study on thermal conductivity of stabilized Al2O3 and SiO2 nanofluids and their hybrid. Int. J. Heat Mass Transfer.

[cit13] Zheng L., Niu J., Zhang X., Ma L. (2012). Dual solutions for flow and radiative heat transfer of a micropolar fluid over stretching/shrinking sheet. Int. J. Heat Mass Transfer.

[cit14] Mahapatra T. R., Nandy S. K., Pop I. (2014). Dual solutions in magnetohydrodynamic stagnation-point flow and heat transfer over a shrinking surface with partial slip. J. Heat Transfer.

[cit15] Rostami M. N., Dinarvand S., Pop I. (2018). Dual solutions for mixed convective stagnation-point flow of an aqueous silica–alumina hybrid nanofluid. Chin. J. Phys..

[cit16] Mousavi S. M., Rostami M. N., Yousefi M., Dinarvand S., Pop I., Sheremet M. A. (2021). Dual solutions for Casson hybrid nanofluid flow due to a stretching/shrinking sheet: a new combination of theoretical and experimental models. Chin. J. Phys..

[cit17] Asjad M. I., Sarwar N., Hafeez M. B., Sumelka W., Muhammad T. (2021). Advancement of non-newtonian fluid with hybrid nanoparticles in a convective channel and prabhakar's fractional derivative—analytical solution. Fractal Fract..

[cit18] Krishna M. V., Ahamad N. A., Chamkha A. J. (2020). Hall and ion slip effects on unsteady MHD free convective rotating flow through a saturated porous medium over an exponential accelerated plate. Alexandria Eng. J..

[cit19] Muhammad K., Hayat T., Alsaedi A. (2021). Heat transfer analysis in slip flow of hybrid nanomaterial (ethylene glycol + Ag+ CuO) *via* thermal radiation and Newtonian heating. Waves Random Complex Media.

[cit20] Krishna M. V., Chamkha A. J. (2021). Hall and ion slip effects on magnetohydrodynamic convective rotating flow of Jeffreys fluid over an impulsively moving vertical plate embedded in a saturated porous medium with ramped wall temperature. Numer. Methods Partial Differ. Equ..

[cit21] Yasir M., Sarfraz M., Khan M., Alzahrani A. K., Ullah M. Z. (2022). Estimation of dual branch solutions for Homann flow of hybrid nanofluid towards biaxial shrinking surface. J. Pet. Sci. Eng..

[cit22] Krishna M. V., Chamkha A. J. (2019). Hall and ion slip effects on MHD rotating boundary layer flow of nanofluid past an infinite vertical plate embedded in a porous medium. Results Phys..

[cit23] Krishna M. V., Swarnalathamma B. V., Chamkha A. J. (2019). Investigations of Soret, Joule and Hall effects on MHD rotating mixed convective flow past an infinite vertical porous plate. J. Ocean Eng. Sci..

[cit24] Krishna M. V., Ahamad N. A., Chamkha A. J. (2020). Hall and ion slip effects on unsteady MHD free convective rotating flow through a saturated porous medium over an exponential accelerated plate. Alexandria Eng. J..

[cit25] Alkasasbeh H. (2022). Numerical solution of heat transfer flow of Casson hybrid nanofluid over vertical stretching sheet with magnetic field effect. CFD Lett..

[cit26] Sun Y., Zheng S. (2020). Influence of particle rotation and partial irradiation on the particle heating up process. Int. Commun. Heat Mass Transfer.

[cit27] Alkasasbeh H., Al Faqih F. M., Shoul A. S. (2023). Computational Simulation of Magneto Convection Flow of Williamson Hybrid Nanofluid with Thermal Radiation Effect. CFD Lett..

[cit28] Yasir M., Khan M., Alqahtani A. S., Malik M. Y. (2023). Numerical study of axisymmetric hybrid nanofluid MgO-Ag/H2O flow with non-uniform heat source/sink. Alexandria Eng. J..

[cit29] Wang C. Y. (1990). Liquid film on an unsteady stretching sheet. Q. Appl. Math..

[cit30] Khan M., Khan U. (2018). Stability analysis in the transient flow of Carreau fluid with non-linear radiative heat transfer and nanomaterials: critical points. J. Mol. Liq..

[cit31] Dzulkifli N. F., Bachok N., Yacob N. A., Md Arifin N., Rosali H. (2018). Unsteady stagnation-point flow and heat transfer over a permeable exponential stretching/shrinking sheet in nanofluid with slip velocity effect: a stability analysis. Appl. Sci..

[cit32] Bakar N. A. A., Bachok N., Arifin N. M., Pop I. (2018). Stability analysis on the flow and heat transfer of nanofluid past a stretching/shrinking cylinder with suction effect. Results Phys..

[cit33] Yahaya R. I., Md Arifin N., Mohamed Isa S. S. P. (2018). Stability analysis on magnetohydrodynamic flow of Casson fluid over a shrinking sheet with homogeneous-heterogeneous reactions. Entropy.

[cit34] Ismail N. S., Arifin N. M., Nazar R., Bachok N. (2019). Stability analysis of unsteady MHD stagnation point flow and heat transfer over a shrinking sheet in the presence of viscous dissipation. Chin. J. Phys..

[cit35] Waini I., Ishak A., Pop I. (2019). Unsteady flow and heat transfer past a stretching/shrinking sheet in a hybrid nanofluid. Int. J. Heat Mass Transfer.

